# Design and implementation of electronic health record-based tools to support a weight management program in primary care

**DOI:** 10.1093/jamiaopen/ooae038

**Published:** 2024-05-13

**Authors:** Polina V Kukhareva, Charlene R Weir, Maribel Cedillo, Teresa Taft, Jorie M Butler, Elizabeth A Rudd, Jesell Zepeda, Emily Zheutlin, Bernadette Kiraly, Michael Flynn, Molly B Conroy, Kensaku Kawamoto

**Affiliations:** Department of Biomedical Informatics, University of Utah, Salt Lake City, UT 84108, United States; Department of Biomedical Informatics, University of Utah, Salt Lake City, UT 84108, United States; Department of Internal Medicine, University of Utah, Salt Lake City, UT 84132, United States; Department of Biomedical Informatics, University of Utah, Salt Lake City, UT 84108, United States; Department of Biomedical Informatics, University of Utah, Salt Lake City, UT 84108, United States; Department of Internal Medicine, University of Utah, Salt Lake City, UT 84132, United States; George E. Wahlen Department of Veterans Affairs Medical Center, Geriatrics Research and Education Center (GRECC), Salt Lake City, UT 84148, United States; Department of Biomedical Informatics, University of Utah, Salt Lake City, UT 84108, United States; Department of Internal Medicine, University of Utah, Salt Lake City, UT 84132, United States; Department of Internal Medicine, University of Utah, Salt Lake City, UT 84132, United States; Department of Family and Preventive Medicine, University of Utah, Salt Lake City, UT 84108, United States; Department of Internal Medicine, University of Utah, Salt Lake City, UT 84132, United States; Department of Pediatrics, University of Utah, Salt Lake City, UT 84108, United States; Community Physicians Group, University of Utah Health, Salt Lake City, UT 84102, United States; Department of Internal Medicine, University of Utah, Salt Lake City, UT 84132, United States; Department of Biomedical Informatics, University of Utah, Salt Lake City, UT 84108, United States

**Keywords:** weight management, obesity, coaching, primary care, electronic health records

## Abstract

**Objectives:**

This paper reports on a mixed methods formative evaluation to support the design and implementation of information technology (IT) tools for a primary care weight management intervention delivered through the patient portal using primary care staff as coaches.

**Methods:**

We performed a qualitative needs assessment, designed the IT tools to support the weight management program, and developed implementation tracking metrics. Implementation tracking metrics were designed to use real world electronic health record (EHR) data.

**Results:**

The needs assessment revealed IT requirements as well as barriers and facilitators to implementation of EHR-based weight management interventions in primary care. We developed implementation metrics for the IT tools. These metrics were used in weekly project team calls to make sure that project resources were allocated to areas of need.

**Conclusion:**

This study identifies the important role of IT in supporting weight management through patient identification, weight and activity tracking in the patient portal, and the use of the EHR as a population management tool. An intensive multi-level implementation approach is required for successful primary care-based weight management interventions including well-designed IT tools, comprehensive involvement of clinic leadership, and implementation tracking metrics to guide the process of workflow integration. This study helps to bridge the gap between informatics and implementation by using socio-technical formative evaluation methods early in order to support the implementation of IT tools.

**Trial registration:**

clinicaltrials.gov, NCT04420936. Registered June 9, 2020.

## Background

The prevalence of obesity is increasing nationwide with few successful long-term interventions.^1^ Self-monitoring and coaching are successful strategies for managing obesity, but they require significant time commitment from coaches.^2^^,^^3^ Primary care is an important source of weight management care and support given the large number of individuals who visit primary care clinics.^4^ However, primary care providers (PCPs) lack the time to engage in coaching, motivational interviewing, population management, and other chronic care management tasks.^5^^,^^6^ Expanding the role of staff to include patient coaching is one solution that has received recent attention.^7–10^ Primary care staff includes medical assistants (MAs), registered nurses (RNs), care coordinators, and others. Primary care staff, especially MAs, are a natural choice to increase the capacity of clinics to deliver chronic and preventive care because they are often the first point of contact when patients arrive in clinic, their time costs less than that of other team members, and they can work on teams with PCPs. As a result, their roles have expanded substantially in recent years with the goal of having them work at the top of their license.^11^

However, there are various implementation barriers to introducing coaching interventions into primary care using existing clinical staff, including staff concern with increased burden and lack of training.^7–10^ The challenges of leveraging primary care staff to deliver coaching has been reported in many contexts, such as diabetes coaching,^12^ medication reconciliation,^13^ and chronic care.^14^ When primary care staff coaching was found to be successful, the participating coaches either had a bachelor’s degree requirement with 2 days/week reserved for coaching^15^ or were hired as full-time coaches.^16^ Challenges with identification and recruitment of eligible patients were also noted as barriers to successful implementation of clinical programs.^17^

Therefore, there is a need for designing and implementing better solutions to support weight management coaching programs, including support of expanded staff roles. It has been demonstrated that implementation science—the study of theories,^18^ validated methods,^19^ and strategies^18^^,^^20^ that facilitate widespread utilization of evidence-based practices—can be successfully used to address barriers to the implementation of obesity treatment.^21^^,^^22^ A combination of implementation science and information technology (IT) tools can be used to support intervention implementation, facilitate patient recruitment,^23^ and enable primary care staff with more limited time allocated to coaching (eg, as an add-on to their usual work duties) to still be able to effectively coach patients.^24–26^ Staff-enabling IT tools include prompts to measure weight, add overweight or obesity to the problem list, and identify patients eligible for weight management programs.^24–26^ Major challenges to designing such IT tools include understanding the workflows involved and the attitudes of providers and staff. Another major challenge is enabling scalable deployment of IT tools through standards-based interoperability. In the recent years, the introduction of new interoperability standards such as the Health Level Seven International (HL7) Clinical Decision Support (CDS) Hooks standard have allowed the creation of novel IT tools that could be more easily deployed across healthcare systems.^27^

To support clinic staff’s expanded roles, this study focused on designing a complex, multi-faceted, interoperable IT approach for a primary care weight management program. Such a complex IT approach required an expansive socio-technical evaluation to design and implement. To our knowledge, this is the first study to report a mixed method formative evaluation of electronic health record (EHR)-integrated IT tools designed to support a weight management program leveraging primary care staff as coaches.

## Objective

The objective of this study was to design and implement IT tools and tool-use tracking metrics to support a weight management program delivered by existing primary care staff.

## Methods

### Weight management program

The primary care weight management program, which served as the focus of this IT tool design and implementation study, is the Maintaining Activity and Nutrition through Technology-Assisted Innovation—Promoting Real World Implementation (MAINTAIN PRIME) program.^28^ Although some individuals successfully lose weight, many regain their lost weight. Therefore, MAINTAIN PRIME is targeted at patients who have lost 5% of their body weight in the last year with the goal of helping patient maintain healthy intentional weight loss. In MAINTAIN PRIME, eligible patients are educated on weight management, coached by primary care staff, and could track their weight, physical activity levels, and calorie intake. The components of MAINTAIN PRIME have been validated in a prior randomized controlled trial (RCT) called MAINTAIN-pc.^29^^,^^30^ While MAINTAIN-pc used experienced health coaches, MAINTAIN PRIME aims to test the feasibility and sustainability of delivering the weight loss maintenance coaching using existing clinic staff as coaches who do not receive any additional effort support to deliver the coaching intervention.

### Study design

**Figure 1.**
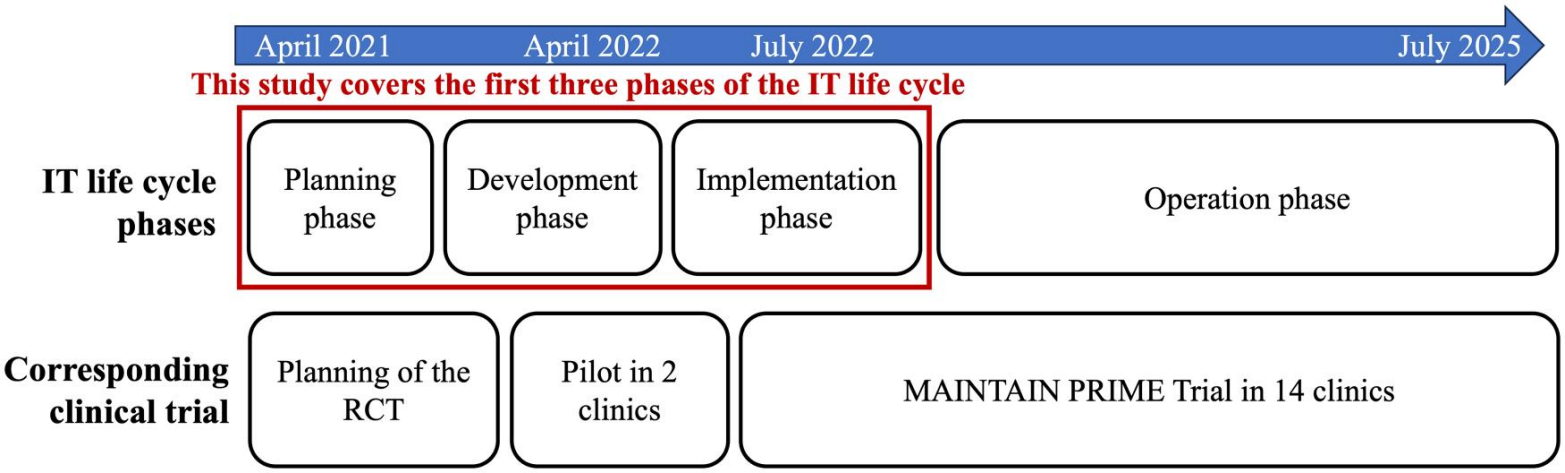
Study timeline in the context of the IT life cycle.

This study focuses on the planning, development, and implementation of IT tools to support the MAINTAIN PRIME program (Figure 1). This study was performed from April 2021 to December 2022 and was informed by a pilot conducted in 2 clinics. The formal MAINTAIN PRIME RCT started in July 2022 and will continue until July 2025. The RCT compares a tracking arm, where patients track their weight, physical activity, and calorie intake in the patient portal, and a coaching arm, which includes both tracking and coaching delivered by primary care staff. The primary outcome of the RCT is patient weight measured by research or clinic staff during research or clinic visits.

This study describes a mixed methods formative evaluation which included (i1) workflow and needs assessment to inform design and workflow integration of IT tools, (2) design of the IT tools, and (3) development of implementation tracking metrics to monitor IT tools use. These evaluations were driven by a socio-technical approach known as the Evaluation in the Life Cycle of Information Technology (ELICIT) framework^31^ that includes a holistic assessment of the context, the users, and the technology involved. Reach, Effectiveness, Adoption, Implementation, Maintenance (RE-AIM) framework was used to guide the formative evaluation and the implementation metrics design.^32^

### Eligible clinics

The program sites included 14 University of Utah primary care clinics with family medicine, internal medicine, and internal medicine/pediatrics specialties. The RCT was preceded by a pilot conducted in 2 out of 14 program clinics. After the pilot, clinics started to join the program one by one at a time convenient for the clinics. The workflow and needs assessments were conducted in 4 program clinics that employed study co-Investigators and/or were ready to start the program. Finally, weekly implementation progress reports monitored by the research team included data from all the clinics that joined the program by the time of the meeting.

### EHR environment

University of Utah uses the Epic EHR. The development of the IT tools was led by a multidisciplinary initiative for interoperable EHR innovations known as ReImagine EHR that specializes in the use of international interoperability standards such as HL7 CDS Hooks.^33^ The governance approval of the EHR-integrated IT tools followed standard governance review processes, including review by the enterprise CDS Committee.

### Eligible coaches

Staff members were eligible to volunteer for coaching in the MAINTAIN PRIME program if they were employed in eligible primary care clinics and received approval to participate from their supervisors.

### Eligible providers

PCPs were eligible to refer their patients to the MAINTAIN PRIME program if they were employed by the eligible clinics. PCPs included attending physicians, residents, physician assistants, and nurse practitioners.

### Eligible patients

Patients were eligible for the MAINTAIN PRIME program if they had intentionally lost 5% of their weight through healthy lifestyle changes in the 2 preceding years and had a pre-weight loss body mass index (BMI) ≥25. Patients were required to be willing to use the patient portal (ie, Epic MyChart) and were offered assistance if they did not have 1. Patients were excluded if they had medical conditions that could cause unintended weight loss or gain, such as cancer, thyroid disease, or edematous states (eg, severe congestive heart failure, end-stage renal disease, or ascites). Patients with bariatric surgery in the last 2 years were also excluded, as well as those planning bariatric surgery or pregnancy in the next 2 years. Patients were also excluded if the PCP’s assessment was that the patient would be unsuitable for the study (eg, if they were unable to safely undertake moderately intense unsupervised physical activity). Finally, patients who lacked basic computer or internet access or were non-English speakers were excluded.

### Project team

The MAINTAIN PRIME research team included 2 principal investigators from primary care and informatics, a clinical trial sub-team, an IT sub-team, a data analytics sub-team, clinical champions, and a socio-technical sub-team. The clinical trial sub-team included an experienced registered dietitian who also acted as project manager and master coach, as well as other registered dietitians and volunteers. This sub-team’s responsibilities included the development of training materials, recruiting, supporting primary care staff as weight management coaches, and evaluation of the coach training. The IT sub-team developed IT interventions and the data analytics sub-team was responsible for data analysis. The clinical champions facilitated coach recruitment, engaged referring providers to support patient recruitment, and advised other sub-teams on clinical operations. Finally, the socio-technical sub-team conducted the analyses described in this manuscript.

### Workflow and needs assessment

Workflow and needs assessments used workflow interviews as well as iterative informal discussion with stakeholders including the project team, administrative leaders, informatics professionals, clinical opinion leaders, and coaches. Data were collected by socio-technical experts and the project manager.

Specific procedures, participants and sample size are described in Table 1. Three types of interviews were conducted, including (1) interviews with MAs and other staff to identify workflow and attitudes toward the added burden of patient recruitment, perceptions of the usability of the participant recruitment tool, and perceptions regarding EHR-based coaching; (2) interviews of clinic administrators to identify perceptions of feasibility and burden of the program; and (3) provider interviews to determine attitudes towards the program, workflow, and perceptions of the usability of recruitment IT tools.^34^

**Table 1.** Sub-study descriptions involved in needs assessment.

Sub-study	Procedures
On-site primary care staff interviews regarding the MA referral process and coaching in 4 study clinics **Sample size**: 24 MAs and 5 RNs	An interview was conducted with semi-structured questions regarding the proposed program, perceived usability of the proposed prompt, usual workflow, and provider-staff communication methods (Supplementary Appendix A).
On-site primary care administrator interviews in 4 clinics **Sample size**: 3 clinic administrators	We presented the MAINTAIN PRIME program to clinic administrators and asked them to describe the clinic approval process and any concerns or suggestions that they had in implementing the program. Usual workflow, communication patterns of providers and staff, staffing concerns and adjustments to COVID were discussed (Supplementary Appendix B).
Provider interviews via Zoom in 4 clinics **Sample size**: 20 providers who had (1) relatively high rates of referrals to dietitians; (2) identified as opinion leaders; and (3) were active in already existing wellness programs were selected.	A 1:1 interview using a critical incident approach with semi-structured questions was used. Providers described a specific patient with whom they had a recent weight management conversation, their clinical goals with that patient, information needs, and usual data collected. The proposed intervention was then presented, and a short usability interview was conducted to review the provider-facing referral prompt prototype (Supplementary Appendix C).

Abbreviations: COVID, coronavirus disease; MA, medical assistant; MAINTAIN PRIME, Maintaining Activity and Nutrition through Technology-Assisted Innovation – Promoting Real World Implementation; RN, registered nurse.

Interviews were recorded and then transcribed. Transcripts were imported into the 12.0 NVivo qualitative analysis software. In the first round of coding, 2 PhD-level socio-technical experts (C.R.W. and T.T.) used an open-ended inductive coding process to independently identify emerging pre-codes.^35^ Second level codes were interactively developed by consensus and discussion after each transcript. Development of the codebook continued until no new codes were identified. The second round of coding consisted of reviewing all quotations within each code and comparing meanings across codes. Codes were aggregated into higher level themes through an iterative process of comparison and discussion by C.R.W., T.T., and J.M.B.

### Design of IT tools to support the MAINTAIN PRIME program

The MAINTAIN PRIME program required 5 functions to be supported by IT tools: (1) identification and recruitment of motivated patients, (2) patient engagement with questionnaires through the patient portal, (3) allowing patients to track their weight, calories, and physical activity through the patient portal, (4) helping coaches keep track of their patients and outstanding tasks, and (5) helping coaches to send evidence-based messages to patients.

The IT tools were selected, designed, and developed by a multi-disciplinary project team. Design of the IT tools for providers, patients, and staff went through multiple iterations using standard user design methodologies including small-scale usability studies and multiple rounds of prototype modifications.^36^ IT tools were tested both formally and informally. Formal testing included review of Epic BestPractice Advisory (BPA) prompts with MAs and providers during the workflow and needs assessment as described in Table 1. Informal usability assessments included regularly seeking feedback from the research team and clinical champions during weekly team meetings. Results of the usability studies were communicated to the director of the ReImagine EHR initiative (K.K.) during weekly team meetings and he later communicated the requirements to the software developers.

### Development approach for IT tools

For patient identification and recruitment, Epic BPA prompts were developed, 1 for providers and another for MAs. The BPA prompts used native Epic functionality (eg, BPA rules) in conjunction with the HL7 CDS Hooks standard. Native BPA rules were used to evaluate for study inclusion and exclusion criteria to identify potentially eligible patients. Furthermore, in order to support providing a graphical summary of the patient’s recent weight loss journey, the HL7 CDS Hooks standard was used to create a dynamic Scalable Vector Graphic (SVG) display, which could be inserted in the context of a CDS Hooks prompt. Such dynamic image generation and display was not possible using native Epic functionality alone. The BPA prompt was then placed in the left-hand patient summary section of the EHR (known as the Storyboard in Epic). A referral order to the study could be placed through the BPA. For the referred patients, study inclusion and exclusion criteria were verified by the research staff during the telephone screening and baseline assessment.

Patient questionnaires were developed using native Epic questionnaire technology. The questionnaire series functionality was used to enable auto-delivery of subsequent questionnaires following completion of prior questionnaires at set time points following patient enrollment in the study. Patient tracking of weight, calories, and physical activity was also implemented using native Epic functionality for capturing patient-entered flowsheet data.

In order to allow coaches to keep track of their patients, an automated EHR participant report was created as follows. First, an Epic registry was created for patients who had been referred to the study. Then, a Workbench Report (a type of interactive report available to end users through the EHR) was created that defined a variety of report Columns that in turn used Extensions to pull in patient information relevant for tracking their status in the study. Similarly, native Epic functionality was used to send evidence-based messages to patients through the definition of message templates using the Epic SmartPhrase technology.

### Specification of automated implementation metrics

In designing implementation metrics, our goals were 3-fold: (1) identify metrics that can be used to inform the implementation process itself on a continuous and automatic basis; (2) ensure that the final implementation outcomes (ie, reach, adoption, implementation fidelity) can be assessed; and (3) support an exploratory analysis of the relationship of implementation outcomes with final clinical outcomes as recommended by Rudd et al.^37^ Two sources of data were used for the metrics: the EHR and a trial-specific REDCap database. This study was informed by the RE-AIM implementation framework.^32^ RE-AIM was used to generate the metrics used for monitoring implementation fidelity, consistency, and impact of adapted changes.

## Results

### Workflow and needs assessment

The findings of the needs assessment are presented in Table 2.

**Table 2.** Workflow and needs assessment results.

Sub-study	Findings
On-site primary care staff interviews regarding the MA referral process and EHR-based coaching	*Theme 1*: Most MAs worked primarily with 1-2 providers in a close-working relationship. Daily huddles occurred with only a few providers. MAs roomed patients, measured vital signs, measured weight and height, performed medication reviews, documented the reason for visit, and reviewed and pended health maintenance needs using protocolized orders sets. They reported doing informal counseling: “When Dr. V comes in, we do a huddle. Every morning, and she lets us know if she wants us to add something to it, or you know, what she wants to add to the previous a planning sheet.” “I’m always with Dr. XX usually but if she’s not here and I’m here that I will help with other providers but when she’s here I’m with her.” *Theme 2*: Workflow is distributed throughout the visit timeline. Review of health maintenance needs, pending orders, and chart review were often done prior to the patient intake, making the alert activation on visit intake too late: “A lot of times patients will talk a lot. And then I completely forget about health maintenance, because it took so long to go through the questions. So, I tried to pend it all (orders) before or just at least review.” *Theme 3*: MAs worry about offending patients by bringing up weight. It might not be typical of the MAs tasks: “But like, if I were to ask a patient, I see that you have lost weight over the two years, I don’t know if they’re gonna take that, like, Do I look sick? Or do I? Did I look fat to you?” “We kind of found that it was something that was better brought up by the physician; patients would get like offended when the MA would talk to them about it and then be completely okay with talking to the physician about it, which happens quite a lot.” *Theme 4*: Referring patients to a new program might be adding a lot of added work: “It just depends on what it entails after the training cause I sort of don’t like it when they keep adding extra things for us to do at check in especially when they tell us they want us to be checking in faster and I’m like what you’re having me review this this this this this this this and asking these additional questions like at first with the care gaps I hated asking those it added like another 5 minutes to my checking in process.” *Theme 5*: MAs and RNs viewed the program as valuable: “We see that weight loss maintenance could be a real benefit to a lot of people. So that’s the reasoning behind talking to them.” *Theme 6*: RNs served as care managers in most clinics. The RNs interviewed reported that coaching was part of their normal scope of practice: “Yes, just because that’s quite often somebody’s health goals, whether they’re diabetic CHF, or you know, so many things. So, it’s kind of a natural fit.” *Theme 7*: MAs (about 30%) showed interest in coaching and felt like it was in their scope of practice. They perceived additional time or support would be needed: “Yeah, they’re usually pretty good about if we have stuff like this, they can find someone to cover me or whatever. So as long as it is scheduled.” “Yeah, um, I don’t do as much online. But I’m sure if [manager name], my manager thought it was good I did. Yeah, no problem.”
On-site primary care administrator interviews	*Theme 1*: Administrators experienced significant stress in maintaining adequate staffing due to COVID, either because there were more MAs out sick, high turnover, or because telehealth visits took more time to set up. However, they expected stress levels to be temporary: “Interviewer: And then do you have a lot of turnover of your medical assistants? and nurses here?” “Manager: Have COVID going on? Yes. A significant amount of burnout. But typically no.” *Theme 2*: Administrators reported a favorable impression of the program and thought that it aligned with the clinic’s mission, but worried about adding another improvement project onto many: “Yeah, yeah. And of course, you want to do the best we can for the patients on everything. So just you know, there’s, there’s always, there’s always room for improvement.” “… there’s usually there’s usually four or five projects that we’re doing all at once. And, and so whenever we’re talking about adding another project, there is a fair amount of fatigue.” *Theme 3*: Using MAs as coaches could cause extra burden as MAs already do many tasks. No prior rules were put in place regarding how and when coaches could work, other than they had to complete the coaching during work time: “Okay, yeah, we might be able to do that for MAs, or, you know, that’s a resource that’s very limited. There’s always downward pressure on reducing the number of MAs that you have a clinic. So, so we don’t have an abundance.”
Provider interviews on Zoom	*Theme 1*: Dealing with weight issues requires persistence as they are common, time consuming, and linked to many other chronic diseases: “I would say, these days, it seems like every at least two or three to eight times a half-day session, so say 20% to 30% of the time because a lot of folks have gained weight within the context of COVID. And so, a lot of folks are finding that their weight is higher than it was last year. And so, it prompts the discussion.” “So, I think it really depends on what is the comorbidity for which they are being evaluated, if it was purely a weight-based discussion, and they don’t have any, they don’t have hypertension, yet, they don’t have diabetes, they don’t have sleep apnea, they don’t really have problems with, you know, terrible back pain or arthritis related complications from obesity, then, you know, we might touch base again, and something between three or four months from our original visit.” *Theme 2*: Tailoring and targeting: strategies to help patients with weight management must be patient-centered, tailored to their personal motivation, and contextualized: “What I’d see is what I’d say with this example is that, um, is that their lifestyle, meaning like their husband works first shift, they work second shift, they’ve got teenagers in the house, there’s so many competing social factors that we’re looking for kind of small gains or small wins…” “And it’s pretty much whatever you choose to do needs to be something that you can live with.” *Theme 3*: Weight management with a patient requires a partnership over time with the whole team involved: “I’ve been caring for her for over a year. So, I see her many times, and we correspond via email and MyChart and all that kind of stuff” “I would say something maybe, you know, closer to a month or so, three weeks or four weeks at the earliest, oftentimes would kind of give them you know, three months, usually, or up to six months to really, to follow up, if on the longest, but usually kind of a two-to -three-month interval… And then that would be an appointment. And yeah, yeah, generally a follow up phone, yeah, follow up appointments. And sometimes they’ll say, you know, keep a weekly log…” *Theme 4*: The MAINTAIN PRIME program could be a valuable addition to the current available tools to help with an important problem: “You know, like, for example, like, that (MAINTAIN) can be a carrot at the end of the stick for a lot of people when you’ve talked about improvements in the blood pressure and diabetes control and things like that improved with, you know, modest weight loss” “Yeah, I have. I certainly have given many referrals for a dietitian, and it is very rarely followed, followed through.” *Theme 5*: Having MAs be the first point of contact about a weight issue should be carefully considered: “I would say I have two very good MAs who’ve been with us a long time… I guess the people who qualify for this have lost weight, which is a good thing. But weight is so emotional. For so many of the patients, I have to say I’m not sure my MAs would feel comfortable with it. And I also think they don’t have time in their flow to do this.” “Sure, I think they could do it with a script.”

Abbreviations: CHF, congestive heart failure; COVID, coronavirus disease; MA, medical assistant; MAINTAIN PRIME, Maintaining Activity and Nutrition through Technology-Assisted Innovation – Promoting Real World Implementation; RN, registered nurse.

### Design of IT tools to support the MAINTAIN PRIME program

The 5 IT tools developed during this project or adapted from MAINTAIN-pc^29^^,^^30^ are summarized in Table 3. The IT tools were designed to minimize impact on clinical workflows and to maximize functionality.

**Table 3.** IT tools developed to support the MAINTAIN PRIME Program.

Intended users	IT tools used	Supported function	Description
Providers and clinical staff	EHR patient referral prompts	Patient identification and patient recruitment conversation	The EHR referral prompts for screening automatically identify eligible patients during the visit. We used staff and provider-facing prompts. See Figure 2A. An EHR referral prompt fires when a potentially eligible patient’s weight is entered into the EHR (usually by the MA during a primary care visit). Referrals may be pended by clinic staff; however, providers must complete and approve all referrals. Research staff conducts enrollment.
Patients	EHR patient questionnaires	Patient engagement with questionnaires through patient portal	Patients complete periodic questionnaires that inquire about their weight management practices.
	EHR flowsheets	Allowing patients to track their weight, calories, and physical activity through patient portal	EHR patient portal flowsheets for tracking weight, calorie intake, and activity levels.
Coaches	Administrative tools	Supporting coaches in managing their workflow and timing messages to patients at the specified intervals	Based on a timeline and patient responses to the questionnaires, coaches provide feedback, evidence-based recommendations, and healthy-lifestyle information through patient portal messages. Administrative tools included EHR participant registry and an intelligent automated Epic Workbench Report (Figure 2B). Reports could be customized by coaches, for example, to filter only for patients who were being coached by them. The reports also included a summary of information on highlighted patients. While the EHR Workbench Report was able to provide much of the information needed to track patients in the study, the EHR tools were insufficient for supporting the rapid identification of all tasks of interest to the coaches and the study team. Therefore, a separate task tracking system (“Task Tracker”) was developed that extracted data from the EHR and identified the tasks that were due at any given time (eg, to encourage a patient who has stopped tracking their weight in MyChart to continue to do so). The initial version of this Task Tracker also included supplemental information, such as the history of MyChart messages between coaches and patients, a summary of patient responses to coaching questionnaires, and a summary of data tracked by the patient in MyChart (eg, calories, steps, weight). The Task Tracker was automatically updated every morning before work hours and made available to coaches as a Microsoft Excel document in a secure Box folder available to all coaches. Based on feedback received from coaches, the Task Tracker was adapted to only include outstanding tasks and associated details, so as to simplify coaches’ interaction with the tool. Further, the Task Tracker was made available in the secure Microsoft Teams workspace for the coaches, which was their preferred location for the tracker.
	Coaching message templates	Composing evidence-based coaching messages	Once coach is ready to write a message, they can use message templates (called “SmartPhrases” in the Epic EHR used in this study) to get started.

Abbreviations: EHR, electronic health record; MA, medical assistant.

**Figure 2.**
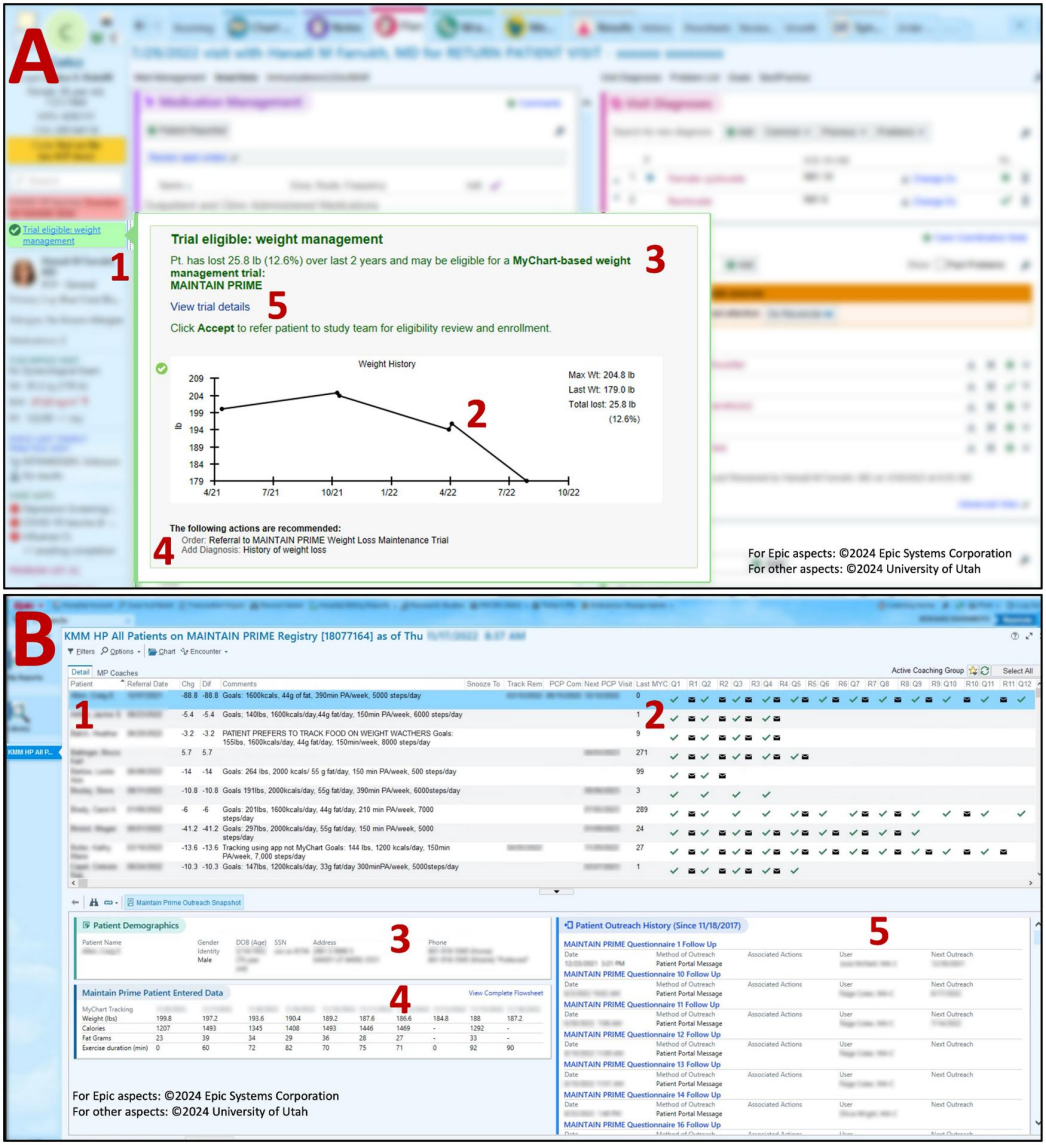
IT tools. (A) Provider-facing recruitment prompt. Note: the prompt includes a weight plot and summary of relevant information about the trial. 1—non-interruptive prompt, 2—weight plot, 3—relevant patient information, 4—referral order, 5—link to trial details for providers who wanted to know more about the study at the time of referral. (B) MAINTAIN PRIME EHR participant workbench report. Note: 1—dashboard overview, 2—status of questionnaire completion and coach responses, 3—detailed patient demographics, 4—patient entered data, 5—patient outreach history.

The **EHR patient referral prompts** were developed based on a user-centered design approach (Figure 2A). Although both providers and staff initially had concerns that discussing weight during the visit may be considered stigmatizing, the referral prompt prototype was well received by all users as the identified patients were likely to be motivated. The full EHR prompts were accessed through hovering over or clicking on the brief summary of the prompt contents on the side panel of the EHR (Item 1 in Figure 2A). The approach used the HL7 CDS Hooks standard to enable visualization of recent weight trends and metrics within the prompt.^38^

Patient weight management tools including EHR patient questionnaires and flowsheets were designed, tested, and validated by MAINTAIN-pc.^29^^,^^30^ The **EHR patient questionnaires** were designed for continuous patient education and engagement. A total of 23 questionnaires covering topics such as on eating out, slips and getting back, and stress management were automatically sent to patients on an automatic schedule, starting with greater frequency initially (weekly) and at a less frequent basis as the patient got established in the coaching program (monthly, then quarterly). The **EHR flowsheets** enabled patients to track their weight, calories, and physical activity through the patient portal.

Coaching tools were designed to facilitate population health management and coaching by primary care staff. The **administrative tools** were essential to integrate coaching into staff workflow. We found that the lack of allocated time for coaching was perceived as a barrier by some coaches (see Table 2, Themes 4 and 7 for primary care staff). The EHR participant registry and associated Workbench Report (Figure 2B) was designed to promote successful administration of the complex intervention timelines. The EHR participant Workbench Report pre-assembled relevant information about participants and their progress in the study. However, the EHR participant Workbench Report was still being iteratively improved during the pilot phase and the first year of the study as early assessments found that coaches were potentially spending more time on coaching tasks than originally planned. Therefore, we also supplemented the EHR participant Workbench Report by developing a supplemental daily task list for the coaches that identified outstanding coaching tasks using a software that we named the “Task Tracker” (Table 3).

Finally, the **coaching message templates** were designed to support evidence-based messages. The master coach implemented these as Epic SmartPhrases that could be inserted into coaching messages, along with a guide on when to use which templates in responding to patient answers to the patient portal questionnaires.

### Automated implementation metrics

We created implementation tracking metrics iteratively through research team discussions based on the stakeholder needs assessment findings in the context of the RE-AIM framework (Table 4).^39^ These implementation metrics were designed to continuously inform the implementation process and to support nimble adjustments as needed. A subset of these metrics was included in weekly reports monitored by the research team, while others were reviewed on a less frequent basis. Ongoing implementation metrics informed several decisions during program implementation. For example, learning that MAs were not effective in pre-placing trial referrals potentially because they often finish care gaps before the visit (and before the weight-triggered prompts fire), led to focusing the efforts on engaging PCPs in the referral process. We also determined that only a few PCPs were actively referring and that we needed to redesign implementation strategies to promote referrals. Overall, implementation metrics were instrumental to timely completion of study enrollment.

**Table 4.** IT implementation metrics using RE-AIM constructs.

IT tools	RE-AIM category	Metric category description	Metric examples
EHR patient referral prompts	Adoption	Providers adopt the program when they refer some proportion of their eligible patients	Percentage of providers referring at least 1 patient Percentage of staff members pre-placed referrals for at least 1 patient
	Reach	The degree to which eligible patients are identified, agree to participate, referred, and eventually enrolled	Percentage of eligible patients referred Percentage of referred patients enrolled in the study
EHR patient questionnaires	Implementation	Patients maintain engagement and participation through completion of the 24 questionnaires throughout the 24 months of the program	Percentage of patients with completed questionnaires across the duration of the program
EHR flowsheets	Implementation	Patients track their weight, calorie intake, and physical activity in the EHR continuously	Percentage of patients who tracked their weight at least once within the first 6 months
Administrative tools	Implementation	Coaches respond to questionnaire submissions and review tracking history in the EHR registry and send message prompts if patients are not tracking	Days between patient completion and coaches’ reply to questionnaire #1 Percentage of patients with completed questionnaire #1 who received a follow up message from a coach within 7 days Percentage of inactive patients (ie, no tracking and no responses to questionnaires) who received a message from a coach within 14 days of becoming inactive
Coaching message templates	Implementation	Coaches send detailed evidence-based high-quality messages	Percentage of messages in which a message template was used

Abbreviation: EHR, electronic health record.

## Discussion

This study highlights the importance of formative evaluation in the design, development, and implementation of IT tools for weight-management interventions in the primary care setting. Four major challenges emerged corresponding to the implementation and workflow integration of the IT tools. First, provider engagement and support are challenging due to busy provider schedules. Feedback to providers about their patients need to be timely, relevant, and easy to utilize quickly. Communication with PCPs was one of the coaching tasks supported by the EHR Workbench Report and Task Tracker. Second, slow patient enrollment in the trial presented a challenge, especially during the COVID-19 pandemic. EHR patient referral prompts were instrumental in supporting recruitment. Third, EHR-based coaching requires intensive IT support of coaches and continuous monitoring of workflow processes. IT tools that support EHR-based coaching are essential and need to be rapidly adapted to the identified usability issues to reduce the negative experiences. Finally, tracking implementation processes can be challenging. The implementation tracking metrics in this study were designed to use existing EHR data, be fully automated, be used weekly and to support a nimble, flexible, facilitative approach that is tailored to the individual clinic. Our results demonstrated that using uncompensated staff as coaches could be feasible when supported by intensive multi-faceted IT tools.

One of the overarching themes in this manuscript is the need for more informatics specific to implementation science research. Although implementation researchers have identified a wide variety of constructs predicting success of health innovations either from empirical research^40^ or from prior theoretical frameworks,^41^ neither approach includes a substantial number of informatics specific constructs (eg, workflow assessment, needs assessment, usability testing, use of interoperability standards). Integrating informatics constructs into implementation science research can inform traditional implementation theories by added depth of the informatics constructs in implementation science research throries.^42^ The results of this work will inform the growing intersection of informatics and implementation science.

The key innovative elements of this study include using an advanced EHR prompt while the patient is still in the clinic to identify patients who lost 5% of their weight and to provide a graphical overview of the patient’s weight journey. A recent review identified only 13 studies describing use of the EHR for clinical trial recruitment, with only 7 of these studies using an alert system and none using real-time identification of patients who lost a specific proportion of their weight.^23^ Moreover, our prompts used the novel HL7 CDS Hooks standard^27^ that allowed visualizing a graphical summary of the patient’s weight; to our knowledge, such use of CDS Hooks for research recruitment has not been described before in the literature. Likewise, the use of an intelligent automated EHR participant report is a novel approach to managing tasks across the patient population.^43^ This is also the first publication to describe the Task Tracker, a newly developed software application that used a clinical database to analyze and extract outstanding coaching tasks in auto-generated spreadsheets, therefore going beyond native Epic EHR functionality to identify and communicate coaching tasks. Furthermore, integrating informatics with implementation science to the degree we demonstrated in this study is innovative because it enriches both areas of scientific inquiry and is critical to informing the results of a pragmatic trial. Workflow analysis, user needs assessment, and iterative user centered design not only informed early design efforts but were instrumental in understanding the key implementation questions of recruitment and coaching. Finally, the innovative expansion of the RE-AIM framework to create automated implementation metrics that are tightly coupled to the intervention itself aided the evaluation process and informed the complex and iterative implementation process.

United States is experiencing physician and clinical staff shortages. Forecasts for the United States anticipate a significant deficit in the medical workforce, specifically a gap ranging from 37 800 to 124 000 physicians over the next 12 years.^44^ The purpose of this manuscript is to outline the capacity of IT solutions, particularly those built upon EHR systems, to bolster clinical staff in their pursuit of practicing at the top of their license. As the shortage of physicians continues, the need for IT-enabled strategies supporting clinical teams to provide even greater patient-centered services with existing resources, such as the one presented in this manuscript, will be increasingly essential. Given the challenges of adding coaching tasks to the responsibilities of already busy clinical staff, we are actively exploring further opportunities for IT-enabled efficiency. For example, we are exploring whether large language models such as ChatGPT could be used to draft messages for coaches.^45^

### Limitations

This study has several limitations. We did not interview patients directly for this study. However, we have implemented processes in place to track their feedback later in the program. Secondly, we conducted this study at one health system. However, this study is implemented in 14 different clinics. Thirdly, the study used a single EHR system. Thus, more research is needed on how to apply the principles in this study to other clinical settings using other EHR platforms. Finally, only English-speakers were included due to resource limitation. However, we are working on expanding the program to include Spanish speakers.

Other limitations emerge from the inherent tradeoffs in a pragmatic, practice-based trial. Providers may have viewed the referral prompt as an alert to remind them to be part of a study, and not necessarily to support patient care. Finally, while study used EHR flowsheets, automatic data uploads from devices (eg, through bluetooth-enabled scales and activity trackers) were not implemented due to resource limitations but would be a natural area for enhancement to explore in the future.

## Conclusions

Developing a sustainable coaching program in primary care is critical to almost all weight management interventions. Given our current lack of understanding regarding interventions that involve interprofessional care in primary care, this mixed methods formative evaluation approach has the possibility of generating sustainable and effective methods for future studies that seek to expand the role of existing clinical staff to assist patients engaged in weight management using IT tools. Moreover, the study bridges the gap between informatics and implementation science by merging theories and methods from both.

## Supplementary Material

ooae038_Supplementary_Data

## Data Availability

The datasets generated and/or analyzed during the current study are not publicly available due private nature of the tracked data and coaching messages but are available from the corresponding author on reasonable request.

## Trial registration

clinicaltrials.gov, NCT04420936. Registered June 9, 2020, https://clinicaltrials.gov/ct2/show/NCT04420936.
