# Supplementary material for: Design and implementation of electronic health record-based tools to support a weight management program in primary care
Source: JAMIA Open. 2024 May 13;7(2):ooae038. doi: 10.1093/jamiaopen/ooae038 (PMC11091423; doi:10.1093/jamiaopen/ooae038)
Supplement: ooae038_Supplementary_Data [file ooae038_supplementary_data.docx]

# Supplementary Materials

**Appendix A. Interview Script for On-site Primary Care Staff**

Part I: Workflow Evaluation

Staff Example Questions

1. How long have you been an MA?
2. Do MAs always follow the same workflow?
3. How are MAs assigned to patients?
4. Are MAs attached to a provider or do they float?
5. Describe the usual workflow after a patient is checked in? (e.g., how do you know the patient is here?)
6. When is the pts. weight entered in the chart?
   1. Who does that?
7. Is there ever a time when you don’t weigh the patient?

Part II: Usability Feedback

Explain the Maintain Prime program to the participant

Show the paper mock-up of the referral alert with script to the participant.

Say: “An alert similar to this will pop-up on the screen when the MA enters the patient’s weight if the patient has lost 5% or more of their highest weight in the last 2 years.”

Say: “The patient’s physician will see the unsigned order in the patient’s chart and will be able to sign the referral order so the patient can be contacted about participating in the study or the physician can cancel the order.”

1. How well would this alert fit in your clinical workflow?
2. How would you feel about notifying patients about the program?
3. Are you interest in being a coach?
4. How would this job fit into their advancement opportunities?
5. When do you have breaks in your workflow that would allow time for coaching?

**Appendix B. Interview Script for On-site Primary Care Administrator Interviews**

1. What strategies and resources are currently used in the clinic for weight loss and will they relate to success? (e.g. do they refer to a group support, dieticians, etc.).
2. Are any MAs or nurses working from home?
3. How many nurses total and/or on an average day?
4. How many MAs total and on an average day?
5. Is there a lot of turnover in this clinic for MAs or Nurses?

**Appendix C. Interview Script for Provider Interviews on Zoom**

Please answer these questions the best that you can. Keep in mind that there are no right or wrong answers. This interview should take about 30 minutes. You will be asked to describe a recent patient and answer some questions about how you approach patient weight management.

1. Please recall a recent patient over the age of 18 with whom you discussed weight loss strategies and goals. Please take the time to review the patient’s chart and your notes.
2. When you are ready, please give a brief report of the patient as you would for a handoff or report.
3. Thank you. Next, please give a brief timeline of events in your interaction with this patient, beginning with any chart review before the visit.

< Let them describe>

< What was the reason for the visit? (well-check, acute problem)>

<Was this a new or established patient?>

<Where do you go in the chart to review the patient’s information?>

1. *Secondly*, what topics did you discuss during the visit? Do you have a script or usual process for visits?
2. I would like to verify that I understand the timeline. First <*describe event*>, and then <*describe next event*>, then < *describe next event*>, etc. --- Is there anything else?
3. Now, let us talk about the weight loss conversation.
   1. At what point(s) during the above timeline did the issue of the patient’s weight come up?
      1. Who brought it up?
      2. Why was it brought up?
   2. What information did you gather from the patient about their weight loss history & how?
      1. if it was intentional?
      2. the timeframe of the weight loss?
      3. strategies the patient used to lose weight?
      4. physical activity or exercise?
      5. patient’s goals?
   3. How did you determine if the patient was motivated to manage their weight?
   4. Were any goals set?
      1. Do you think the patient goals are reasonable?
         1. Why or why not?
      2. Did you explore diet habits?
      3. How were these decided?
   5. How did you find relevant information?
      1. Where did you look in the patient’s record to review their weight over time?
      2. Where in the medical record are patient’s weight loss strategies documented?
      3. How would you be able to find the patient’s prior weight loss goals in the medical record?
   6. How did you document the goals and/or the patient’s plans?

The remaining questions refer to your clinic’s general practices.

1. **Tracking progress**
   1. How do you track success for a weight management goal?
      1. <How do you remember the goal at the next visit?>
   2. What metrics do you use to define success in weight management?

<For example, goals, rate of change, diet & exercise>

1. **Description of workflow processes**
   1. What are the current practices for providing weight loss support in your clinic?
   2. What barriers do you perceive in supporting weight loss management at your clinic?
   3. What strategies have you found to be effective in helping patients manage their weight?
   4. Do care managers regularly work with patients on issues of weight management?
   5. *Would it be plausible for MAs or Care Managers to provide weight management support, if they received training?*
2. **Shared decision making**
   1. What is your belief about your responsibility for advising patients regarding weight management?
   2. What strategies do you use to actively motivate patients to manage their weight?
   3. If patient education is needed for weight management, how is that provided?
   4. Could you characterize how you set goals with the patient?
   5. How comfortable are you with recommending weight loss management services to a patient?
3. **Provider Preferences**
   1. If the EMR detected that a patient had started losing weight, would you be comfortable with getting a notification requesting that you refer the patient for weight loss maintenance support services?
      1. When would you want to receive the request?
      2. If the notification was in the patient’s chart, where would you most like to see it?
         1. Would a popup alert be acceptable?
   2. Would you want to receive reports of the patient’s progress from the support service?
      1. What specific information would you want?
      2. In what format would you prefer the information? (email, scanned in the patient’s EMR, Note, data in the EMR, pop-up other)
